# Feed intake, growth performance and carcass characteristics of Damara lambs fed bush-based rations from four encroacher bush species

**DOI:** 10.1007/s11250-025-04473-w

**Published:** 2025-06-04

**Authors:** Katrina Lugambo Shiningavamwe, Emmanuel Lutaaya, Johnfisher Mupangwa

**Affiliations:** 1 Ministry of Agriculture, Water and Land Reform, Windhoek, Namibia; 2 Department of Animal Production, Agribusiness and Economics, University of Namibia, Windhoek, Namibia

**Keywords:** Bush encroachment, Average daily gain, Finishing rations, Feed resources, Carcass characteristics

## Abstract

The effect of feeding bush-based finishing rations on the performance of Damara lambs was studied. Thirty weaned lambs weighing 16.7 ± 1.9 kg were allocated to five treatments in a completely randomized design over a 90-day feeding period. The control diet (T1) consisted of Lucerne (10%), grass hay (30%) and concentrate mix (60%). The other diets consisted of roughage (40%) from the milled bushes *Senegalia mellifera* (T2)*, Dichrostachys cinerea* (T3)*, Terminalia sericea* (T4) and *Rhigozum trichotomum* (T5) and concentrate mix (60%). At the end of the feeding trial, the lambs were slaughtered and carcass characteristics were evaluated. The average daily feed intake (ADFI) was affected (*P* < 0.05) by sex, treatment, week and treatment x week interactions. The ADFI for T1 exceeded (*P* < 0.05) that for T2 and T3, at most time points. The ADFI of T1 and T4 were similar (*P* > 0.05) at weeks 6 to 10, but differed (*P* < 0.05) at other time points. The average daily gain (ADG) and feed conversion ratio (FCR) were affected (*P* < 0.05) by sex and treatment. The ADG (g/day) least squares means (± S.E) for T1 – T5 were 148.0 ± 6.9, 156.4 ± 6.9, 124.2 ± 6.9, 133.7 ± 6.9 and 133.7 ± 6.9, respectively. Treatment T2 had a better (*P* < 0.05) FCR compared to other bush-based treatments. Males had heavier (*P* < 0.05) final, hot and cold carcass weights than females. Lambs fed T4 had greater (*P* < 0.05) rib eye area than T1 (8.3 ± 0.5 vs. 5.9 ± 0.5 mm^2^). Bush-based diets can serve as production diets for weaned sheep and result into acceptable weight gain and carcass quality.

## Introduction

Sheep are predominantly raised on rangelands in the arid and semi-arid regions of the subtropics where they are able to utilize marginal pastures. A major constraint to such a sheep production system is the fluctuation in both quantity and quality of the feed supply on rangelands (Olafadehan and Adewumi 2010). Consequently, there is low animal productivity with animals taking longer to reach slaughter weights and often producing lower quality carcasses (Ben Salem et al. 2004). Global climate change is expected to increase the frequency of occurrence of extreme events like droughts further exacerbating the feed shortages in livestock production systems based on rangelands as the main feed resource.

As an alternative, finishing sheep in the feedlot can play an essential role in preparing lambs for slaughter, as well as relieving the grazing pressure on pasture. Mutton and lamb producers in Namibia and South Africa use Lucerne and grass hay as roughage sources in feedlot diets (van der Merwe et al. 2020). However, the limited availability and high cost of these ingredients in the Namibian market often limit their use as roughage sources in feedlot diets. Hence, interventions to find affordable and sustainable alternative roughage sources are important for the sheep industry and the environment.

In Namibia, there is an abundance of encroaching bushes that present an opportunity to be explored as a possible alternative roughage feed resource for livestock. Honsbein et al. (2018) evaluated the feeding value of *Senegalia mellifera* milled bush as a replacement for grass hay in total mixed feedlot diets of Sanga cattle, which obtained an average daily live weight gain of 880 g/day. To our knowledge, there is paucity of information on the feeding value of rangeland encroacher bush species, as alternative roughage fodder to the conventional feed like grass and Lucerne hay for ruminant livestock. In addition, it is also necessary to test performance of other ruminant species like sheep when finished on diets containing milled bush as a roughage source. Hafez (1987) for instance reported that sheep were more efficient in converting fibrous low-quality feedstuffs into meat and other livestock products than cattle. It was hypothesized that milled encroacher bushes of *Senegalia mellifera*, *Dichrostachys cinerea*, *Terminalia sericea* and *Rhigozum trichotomum* could be used as suitable alternative roughage source, for growing animals and may improve the overall performance of sheep. Therefore, the objective of this study was to evaluate the effects of feeding the four encroacher bush species as alternative roughage source on the feed intake, growth performance and carcass characteristics of Damara lambs.

## Materials and methods

The feeding experiment was conducted at the Neudamm Campus of the University of Namibia, approximately 30 km east of Windhoek in the Khomas region of Namibia. The campus is situated at 22°30′10.19"S latitude and 17°22′5.39"E longitude (Mendelsohn et al. 2002). All procedures conducted during this experiment were approved by the Animal Research Ethics Committee (ref: AREC/024/2020) of the University of Namibia. The approval process for research ethical consideration involved submitting a full research proposal and all relevant documents to the AREC structures. The evaluation considered aspects of animal health, behaviour and welfare and protection of the environment. The committee examined the housing environment, general management, potential harmful effects of the treatment and measures to mitigate any adverse effects e.g. should animals fall sick. The application went through the evaluation process including site visit to ensure compliance with ethical principles and values and that the research abided by the ethical committee principles of the Declaration of Helsinki. The committee ensured that any shortcomings of the proposal were addressed by the research team before approval was granted.

### Feed ingredients and experimental diets

The milled bush biomass of encroacher bush species used in this study was of the predominant encroacher species, namely *S. mellifera, D. cinerea*, *T*. *sericea* and *R*. *trichotomum.* Harvesting of the bushes was done between April and May 2019 and the harvested biomass was restricted to branches or twigs of ≤ 20 mm stem diameter. The fresh biomass was milled using a hammer mill to a particle size of 10 mm and air dried under shade until constant weight before being packed in bags and transported to Neudamm campus for storage until the feeding trial.

Apart from bush biomass, other roughage sources used in the preparation of experimental diets were grass hay (mixed veld grass species) and Lucerne hay, which were also milled to particle sizes of 10 mm. The experimental diets were formulated to constitute 40% of different roughage sources and a similar 60% concentrate made from a combination of different feed ingredients (Table 1). Except for bush biomass, all other ingredients were purchased from a commercial feed supplier. A conventional diet consisting of coarsely ground Lucerne (10%) and grass hay (30%) as roughage, was used as a control (T1), while each of the other four treatment diets (T2-T5) consisted of the selected bush species as roughage source at the same inclusion rate (40%) and the remaining portion (60%) consisted of the same combination of supplements. The control was designed with current farmer practice in mind, where they aim at meeting growing sheep maintenance requirements and a modest gain, particularly during the drought years. Batches of feed were mixed weekly and fed to the lambs. All treatment diets were formulated to meet the nutrient requirements of growing sheep for protein (140 g/Kg, CP) and energy (9 MJ/Kg, ME) according to NRC (2007) recommendations.

**Table 1:** Ingredients composition of the five treatment diets

Feed ingredient (kg “as is”)	Treatment diets*				
	T1	T2	T3	T4	T5
Coarsely ground grass hay	30	0	0	0	0
Coarsely ground lucerne hay	10	0	0	0	0
Milled Bush	0	40	40	40	40
Yellow maize meal	22	20	20	19	20
Molasses syrup	5	5	5	5	5
HPC 30	30	32	32	33	32
Futterfos™ P14	1.5	1.5	1.5	1.5	1.5
Coarse salt	1.5	1.5	1.5	1.5	1.5
Total	100	100	100	100	100

* *T1* control diet, *T2 Senegalia mellifera-*based diet, *T3 Dichrostachys cinerea*-based diet, *T4 Terminalia sericea-*based diet and *T5 Rhigozum trichotomum*-based diet; *HPC 30* high protein concentrate with 30% crude protein; *Futtterfos™* P14 Phosphate lick with 14% Phosphorus

### Experimental animals and management

Thirty (30) weaned 3-month old Damara lambs (15 males and 15 females) with a mean weaning weight of 16.7 ± 1.9 kg were used. The lambs were vaccinated with Multivax P™ (Intervet International B.V., The Netherlands) at the beginning of the trial to protect them against botulism, black quarter, pulpy kidney and clostridium. They were also treated with Dectomax™ (Pfizer Laboratories (Pty) Ltd, Sandton) against internal and external parasites.

### Experimental design and data collection

The experiment was set up as a completely randomized design (CRD) with six (6) lambs per treatment (3 males and 3 females), to evaluate feed intake (FI) and the growth performance for 90 days, after an adaptation period of 14 days. The 30 lambs which had been grazing extensively on range were randomly allocated using a table of random digits to the 5 dietary treatments. Lambs were housed individually in pens of 1 m^2^ with concrete floors in an open-sided roofed shed with natural ventilation, with summer month temperatures ranging between 20—35 °C, where they received treatment diets and water on an *ad lib* basis.

The lambs were weighed weekly on the same day of the week in the morning before feeding, until the end of the experiment. After the adaptation period, the feed offered and feed refused daily were weighed and recorded to determine the feed intake. The daily feed was offered to each sheep in two portions, at 09 h00 and 14 h00. Initial body weights were obtained by weighing the lambs using an electronic scale (Micro T7E Scale; Premier Scale Services (Pty) Ltd) at the beginning of the adaptation period. Average daily gain (ADG) was calculated as the difference between final and initial body weights divided by the number of feeding days.

### Sampling and chemical composition analysis of experimental diets

During the trial period, random samples of each treatment diet were taken once from a weekly batch, mixed and pooled separately in marked paper bags. At the end of the experiment period, all treatment diet samples were ground to pass through a 1 mm sieve (Retsch Mable mill; Retsch GmbH) and stored in plastic bottles for chemical analysis. The dry matter (DM) content of bush species samples was determined by drying the samples in a forced draught oven at 100 °C for 24 h (AOAC 2000). Ash was determined by incineration in a muffle furnace at 550 °C for 6 h (AOAC 2000). The crude protein (CP) method no. 978.04 (AOAC 2005) was used to determine the total nitrogen content and CP was estimated by multiplying the percentage of N content by a factor 6.25. Ether extract (EE) was determined using the AOAC method 920.39 (AOAC 2000). Ash-free neutral detergent fibre (NDFom) and Ash-free acid detergent fibre (ADFom) were determined following the procedures of Mertens (2002) with NDFom assayed without use of an alpha-amylase, but with sodium sulfite. Ash-free Acid detergent lignin (ADLom) was determined by placing dried ADF samples in 72% sulphuric acid (H_2_SO_4_) (Mertens 2015). Calcium and Phosphorus were determined using inductively coupled plasma atomic emission spectrometry (Sah and Miller 1992). Table 2 shows the chemical composition of the five diets used in the trial.

**Table 2:** Chemical composition (g/kg DM) of the five treatment diets

	Treatment diets*						
Variables	T1	T2	T3	T4	T5	SEM	*P*-value
Dry matter (DM)	910^b^	903^c^	918^a^	890^d^	904^c^	1.04	< 0.01
Organic matter (OM)	869^c^	890^b^	904^a^	869^d^	885^b^	1.07	< 0.01
Crude protein (CP)	146^a^	143^ab^	135^c^	140^bc^	139^bc^	1.53	0.03
Neutral detergent fibre (NDFom)	415^b^	422^b^	506^a^	386^c^	386^c^	5.12	< 0.01
Acid detergent fibre (ADFom)	221^d^	226^b^	365^a^	245^c^	240^c^	3.37	< 0.01
Acid detergent lignin (ADLom)	44.3^c^	73.3^b^	93.3^a^	93.3^a^	84.1^ab^	3.55	< 0.01
Ether extract (EE)	16.0^d^	16.8^c^	19.3^a^	20.0^a^	17.5^b^	0.16	< 0.01
Ash	131^a^	110^c^	96^d^	131^a^	115^b^	1.07	< 0.01
Calcium (Ca)	9.3^c^	11.5^b^	9.6^c^	13.2^a^	9.7^c^	0.27	< 0.01
Phosphorus (P)	0.507^c^	0.475^d^	0.410^e^	0.570^a^	0.530^b^	0.002	< 0.01
Metabolizable energy (MJ/kg)^1^	9.3	9.9	9.6	9.1	9.8	N/A	N/A

* *T1* control diet, *T2* = *Senegalia mellifera-*based diet, *T3 Dichrostachys cinerea*-based diet, *T4 Terminalia sericea*-based diet and *T5 Rhigozum trichotomum*-based diet

^1^Calculated metabolizable energy (ME) values based on the feed formulation program; *SEM* Standard error of means; N/A = not applicable; ^**a−d**^Means with different superscripts within a row differ **(***P* < 0.05**)**

### Slaughtering procedures

At the end of the feeding experiment, all sheep were individually weighed to obtain the final weight (FW), after being fasted overnight, with only access to water, to avoid carcass contamination with contents from the digestive system. They were then transported to the Neudamm abattoir for slaughter. Sheep were slaughtered in accordance with the SAMIC (2006) regulations at the registered abattoir facilities of the Neudamm campus, under the required conditions for local abattoirs in Namibia. Animals were electrically stunned to render them unconscious and to ensure that they did not suffer pain during slaughter. The lambs were slaughtered humanely to facilitate exsanguination. The carcasses were hanged on the bleeding rail and the dressing operation began after the bleeding process was completed. All slaughtering procedures were done under the supervision of a veterinarian, specialized in Veterinary Public Health.

### Carcass characteristics

After dressing and evisceration procedures, carcass grading was done visually, by a qualified carcass grader, according to the South African Meat Classification System for beef, lamb, mutton and chevon, according to the Government Notice No. R.863 of 1 September 2006 (Republic of South Africa 2006), which is also adopted by the Meat Board of Namibia. The carcasses were classified based on the age and carcass fatness (subcutaneous fat cover/score). The carcasses were weighed to obtain hot carcass weights (HCW). The pH of the carcasses was measured 1 h (pH_1_) after slaughter in the left *Musculus longissimus dorsi* at the area between 10 to 13th rib, using a portable digital pH meter (pH5 Tester Kit (IP67), XS Instruments, Italy) with a penetration electrode and temperature probe. Subsequently, carcasses were chilled in a cooler under the temperature of 2–4 °C for 24 h and weighed to obtain the cold carcass weights (CCW). Cold carcass weight (CCW) was expressed as a proportion of the live weight at slaughter (SW) to determine the dressing percentage (Van der Merwe et al. 2020). The pH reading of the carcasses was taken again at 24 h (pH_24_) in the *Musculus longissimus dorsi* on the same spot where pH_1_ was taken. After 24 h of chilling, the carcasses were then split into two halves down the spinal column by a longitudinal cut on the vertebral column using a meat band saw. The left side of the carcass was used for further measurements such as external length (CEL) of each carcass, shoulder and buttock circumferences (SC and BC, respectively), which were measured with a flexible tape measure following the procedure described by De Boer et al. (1974).

The fat thickness was measured at ¼ (Fat Pos¼), ½ (Fat Pos½) and ¾ (Fat Pos¾) positions on the 12th rib from the chine bone end, with a vernier calliper and from these, average fat thickness was calculated (Lima et al*.* 2015). The eye-rib depth and the eye-rib width of the *Musculus longissimus dorsi* exposed by cutting the carcass between the 12th and 13th ribs were measured with a Vernier calliper (Ferreira et al. 2012; Landim et al 2015). As per the method described by Ferreira et al. (2012), the area of *Musculus longissimus dorsi* was determined by measuring the maximal width of the muscle, denoted as “A” and its maximal depth “B” and then applying the values into the following equation: (A/2 × B/2) × , where equals 3.1416. The three-rib cut was weighed and physically separated by blunt dissection into bone, lean and fat tissue which were weighed individually and expressed as a proportion of the cut as described by Van der Merwe et al. (2020).

### Statistical analysis

The chemical composition of the diets was subjected to analysis of variance (ANOVA) using Proc GLM (SAS 2009). Similarly, FCR and ADG were subjected to analysis of variance (ANOVA) using Proc GLM (SAS 2009) with the effects in the model being sex and treatment.

Intake is a function of the body weight (W) of the animal, hence analyses were based on metabolic body weight (W^0.75^). The model (1) included the effects: sex, treatment, week and treatment x week interactions. Data was analysed using Proc Mixed (SAS 2009) which considers correlation between repeated measures on an individual and the Bayesian Information Criterion (BIC) which compares covariance structures based on goodness of fitness criteria, was used to select the appropriate covariance structure (Littell et al. 1998), which was SIMPLE. Estimate statements were used in Proc Mixed (SAS 2009) to compare means and obtain standard errors. Effects were considered significant at *P* < 0.05.

1 $${\text{Y}}_{\text{ijkl}} = \mu + {\text{F}}_{\text{i}} + {\text{S}}_{\text{j}} + {\text{W}}_{\text{k}} + {\text{FW}}_{\text{ ik}} + {\text{e}}_{\text{ijkl}}$$

where: Y_ijkl_ = feed intake or body weight**;** μ = overall mean**;** F_i_ = effect of treatment (i = T1, T2… T5)**;** S_j_ = effect of sex (j = male, female); W_k_ = effect of week (k = 1,2,3…13)**;** FW _ik_ = interaction effect between treatment and week; e_ijkl_ = random error term explaining variation among experimental units (EU) on the same treatment.

A similar model to (1) was used to fit body weights, but with the additional interaction effect of treatment by sex. The best covariance structure for body weights was ante-dependence [ANTE(1)].

The data on carcass characteristics was subjected to analysis of variance (ANOVA) using the General Linear Model (GLM) procedure (SAS 2009). The effects in the model were treatment (T1-T5), sex and their interactions. Data were checked for normality and transformation was carried out where necessary, before statistical analysis. Means were separated using the Turkeys’ Studentised range test.

## Results

### Feed intake

**Fig. 1:**
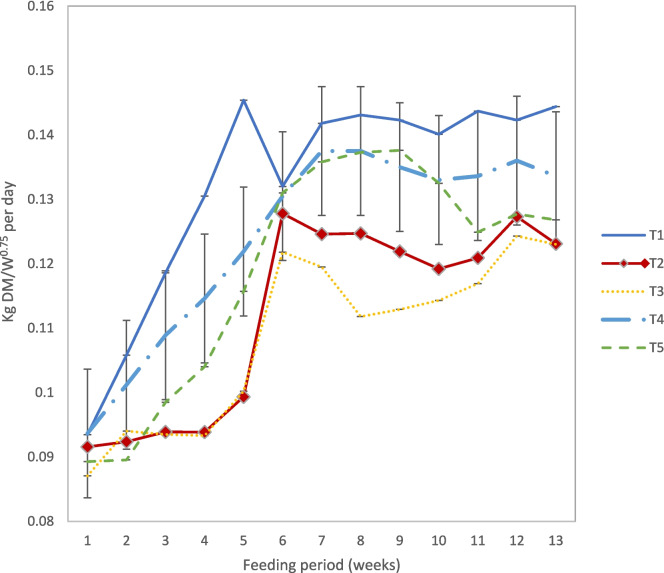
The average daily feed intake (kg DM/kg W^.75^ per day) of weaned Damara sheep. * *T1* control diet; *T2 Senegalia mellifera-*based diet; *T3 Dichrostachys cinerea*-based diet; *T4 Terminalia sericea-*based diet and *T5 Rhigozum trichotomum*-based diet

The average daily feed intake (ADFI) of the lambs (kg DM/kg W^0.75^per day) during the ninety (90) days feeding period was affected (*P* < 0.05) by sex, treatment, week and treatment * week interaction (Fig. 1). Averaged over the trial period, the ADFI of T1 exceeded (*P* < 0.05) that of other treatments.

The ADFI generally increased from week 1 (0.91 kg DM/kg W^.75^ per day) by about 41% to week 6—7 (0.129 kg DM/kg W^.75^ per day) and then stabilized, although lowest intake was for T2 and T3, which also showed the greatest lag. The ADFI for T1 exceeded (*P* < 0.05) that for T2, T3 and T5, at most time points. Other than at weeks 3, 4, 5, 11 and 13, ADFI for T1 and T4 did not differ (*P* > 0.05). The ADFI of diets T2 and T3 were similar (*P* > 0.05) except at week 8. The ADFI of T4 exceeded (*P* < 0.05) that of T2 and T3 at most time points. ADFI of T5 exceeded (*P* < 0.05) that of T3 from weeks 4 to 10. The ADFI least squares means (± S.E) over the trial period was: T1 (0.133 ± 0.001), T2 (0.112 ± 0.001), T3 (0.109 ± 0.001); T4 (0.124 ± 0.001) and T5 (0.119 ± 0.001). From estimated contrasts, the ADFI of the control diet appears to have peaked at week 6 where it plateaued. The ADFI for diets T2, T3, T4 and T5 reached their maximum at week 7 where they remained stable. The least squares mean (± S.E) (kg) for ADFI of females exceeded (*P* < 0.05) that for males (0.121 ± 0.001 vs. 0.118 ± 0.001).

### Growth performance

**Fig. 2:**
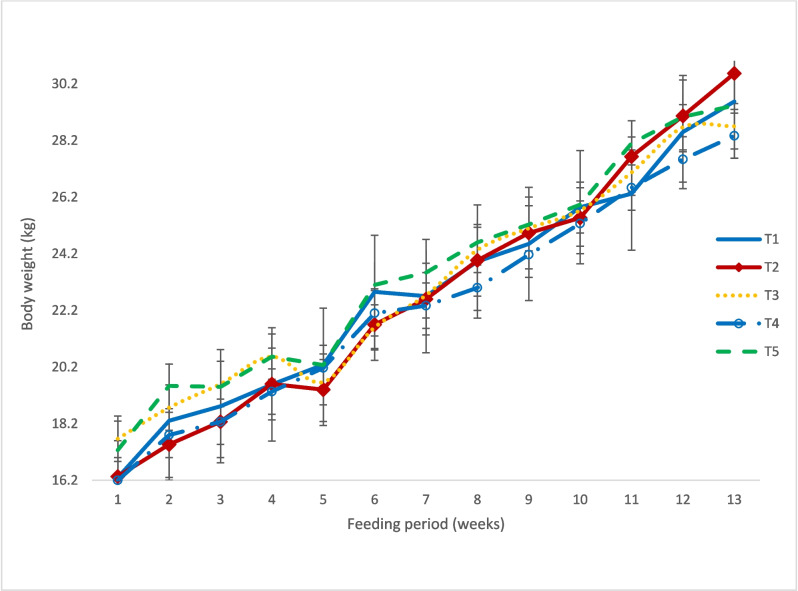
Least squares means of body weight (kg) of Damara lambs during the feeding period. * *T1* conventional feedlot diet, *T2 Senegalia mellifera-*based diet, *T3 Dichrostachys cinerea*-based diet, *T4 Terminalia sericea-*based diet and *T5 Rhigozum trichotomum*-based diet

The live weights were influenced (*P* < 0.05) by week, treatment * week and treatment * sex interactions. Figure 2 shows the least squares means of body weights for lambs on the different diets. The body weights increased linearly, but with fluctuations by diet. Only T2 body weights exceeded (*P = *0.04) those of T4 at week 13. The control diet (T1) did not differ (*P* > 0.05) from other treatments.

The least squares means for growth performance are shown in Table 3. With the exception of diet T4, there were no differences (*P* > 0.05) in live weights between males and females within each treatment diet; females on T4 were heavier (*P = *0.0001) than males. The ADG and FCR were affected (*P* < 0.05) by sex and treatment. Treatment T2 gave higher (*P* < 0.05) ADG than other treatments. The least squares means for ADG (g/day) were 124.9 ± 4.3 for females and 153.5 ± 4.3 for males. Treatment T2 had a better (*P* < 0.05) FCR compared to other bush-based treatments (T3, T4 and T5), but did not differ (*P = *0.098) from the control (T1). The FCR for T3, T4 and T5 were also similar (*P* > 0.05) to the control (T1). The FCR for females was 9.8 ± 0.3 and for males was 8.1 ± 0.3.

**Table 3:** Least squares means (± SEM) of growth variables for Damara lambs fed five diets from different roughage sources

	Treatment diets*						
Variable	T1	T2	T3	T4	T5	SEM	*P*-value
Initial weight (kg)	16.2	16.3	17.7	16.2	17.3	0.77	0.56
Final weight (kg)	29.7	30.6	28.7	28.4	29.4	0.87	0.43
Total gain (kg)	13.5	14.2	11.8	12.2	12.2	0.83	0.24
#Average daily gain (g/day)	148.0^b^	156.4^a^	124.2^b^	133.7^b^	133.7^b^	6.9	< 0.0001
#Feed conversion ratio	8.8^ab^	7.6^b^	9.5^ab^	9.6^a^	9.4^a^	0.5	< 0.0001
#Final body weights (kg) for males	23.4^a^	22.9^a^	23.5^a^	19.8^b^	23.3^a^	1.0	0.01
#Final body weights (kg) for females	22.7^b^	22.8^b^	22.7^b^	25.0^a^	23.8^b^	1.0	0.01

* *T1* control diet, *T2 Senegalia mellifera-*based diet, *T3 Dichrostachys cinerea*-based diet, *T4 Terminalia sericea-*based diet and *T5 Rhigozum trichotomum*-based diet; ^**a−d**^Means with different superscripts within a row differ **(***P* < 0.05**).** # Least squares means; *SEM* Standard error of means

### Carcass characteristics

The final weight (FW) and carcass characteristics results are presented in Table 4. The lambs from all five treatment groups had a visually subcutaneous fat cover of score 1, and they all fell under the same carcass grade A1. Treatment did not influence (*P = *0.09) hot carcass weight (HCW). The FW, HCW and cold carcass weight (CCW) were influenced (*P* < 0.05) by sex. Irrespective of the treatment diet, the FW, HCW and CCW were lighter (*P* < 0.05) for female than male lambs, while the dressing percentage did not differ (*P = *0.09) by sex.

**Table 4:** Slaughter weight and carcass characteristics least squares means (± SEM) of the Damara lambs fed different diets

Variables	Treatment					Sex		P values	
	T_1_	T_2_	T_3_	T4	T5	F	M	Treatment	Sex
Carcass grade	A1	A1	A1	A1	A1				
FW (kg)	29.7 ± 0.9	30.6 ± 0.7	28.7 ± 0.7	28.4 ± 0.7	29.4 ± 0.7	28.0 ± 0.4^b^	30.7 ± 0.4^a^	0.17	< 0.05
HCW (kg)	12.3 ± 0.3	12.3 ± 0.3	11.3 ± 0.3	11.3 ± 0.3	11.5 ± 0.3	11.4 ± 0.2^b^	12.1 ± 0.2^a^	0.09	0.03
CCW (kg)	11.9 ± 0.3	11.8 ± 0.3	10.9 ± 0.3	10.9 ± 0.3	11.0 ± 0.3	11.0 ± 0.2^b^	11.6 ± 0.2^a^	0.11	0.05
CCD (%)	40.0 ± 0.8	38.4 ± 0.8	37.7 ± 0.8	38.4 ± 0.8	37.4 ± 0.8	39.0 ± 0.5	37.8 ± 0.5	0.18	0.09
CEL (cm)	56.7 ± 0.8	54.8 ± 0.8	54.8 ± 0.8	54.0 ± 0.8	54.2 ± 0.8	54.7 ± 0.5	55.1 ± 0.5	0.18	0.65
SC (cm)	21.8 ± 0.4	22.0 ± 0.4	21.2 ± 0.4	20.3 ± 0.4	21.0 ± 0.4	21.1 ± 0.3	21.4 ± 0.3	0.05	0.48
BC (cm)	34.8 ± 0.6	35.2 ± 0.6	35.0 ± 0.6	34.0 ± 0.6	35.2 ± 0.6	34.8 ± 0.4	34.9 ± 0.4	0.67	0.91
pH_1_	5.6 ± 0.1^b^	5.9 ± 0.1^b^	5.8 ± 0.1^b^	5.8 ± 0.1^b^	6.2 ± 0.1^a^	5.8 ± 0.1	5.9 ± 0.1	0.01	0.74
pH_24_	5.3 ± 0.04	5.3 ± 0.04	5.3 ± 0.04	5.3 ± 0.04	5.3 ± 0.04	5.3 ± 0.03	5.3 ± 0.03	0.86	0.28
Fat_1(mm)	3.3 ± 0.5	4.1 ± 0.5	2.6 ± 0.5	2.7 ± 0.5	2.9 ± 0.5	3.5 ± 0.3	2.7 ± 0.3	0.31	0.12
Fat_2 (mm)	2.7 ± 0.4	2.5 ± 0.4	2.0 ± 0.4	2.4 ± 0.4	1.8 ± 0.4	2.3 ± 0.3	2.2 ± 0.3	0.30	0.59
Fat_3 (mm)	2.8 ± 0.4	2.0 ± 0.4	2.2 ± 0.4	3.1 ± 0.4	2.4 ± 0.4	2.7 ± 0.3	2.3 ± 0.3	0.28	0.30
Avg fat (mm)	3.0 ± 0.3	2.8 ± 0.3	2.3 ± 0.3	2.8 ± 0.3	2.4 ± 03	2.9 ± 0.2	2.4 ± 0.2	0.43	0.13
Rib eye-width (cm)	4.4 ± 0.1	4.5 ± 0.1	4.7 ± 0.1	4.7 ± 0.1	4.5 ± 0.1	4.5 ± 0.1	4.6 ± 0.1	0.29	0.51
Rib eye-depth (cm)	1.7 ± 0.1^b^	2.0 ± 0.1^ab^	2.0 ± 0.1^ab^	2.2 ± 0.1^a^	1.9 ± 0.1^b^	2.0 ± 0.1	2.0 ± 0.1	< 0.05	0.63
Rib eye area (cm^2^)	5.9 ± 0.5^b^	7.1 ± 0.5^ab^	7.2 ± 0.5^ab^	8.3 ± 0.5^a^	6.8 ± 0.5^b^	7.1 ± 0.3	7.0 ± 0.1	0.038	0.78

* *T1* control diet, *T2 Senegalia mellifera-*based diet, *T3 Dichrostachys cinerea*-based diet, *T4 Terminalia sericea-*based diet and *T5 Rhigozum trichotomum*-based diet. *FW* Final weight; *HCW* hot carcass weight; *CCW* cold carcass weight; *CCD* cold carcass dressing; *CEL* carcass external length; *SC* shoulder circumference; *BC* buttock circumference; *SEM* standard error of the mean;* F* Female; *M* Male. ^**ab**^Means with different superscripts within a row differ **(***P* < 0.05**)**

None of the treatments significantly (*P* > 0.05) affected: carcass yield; average subcutaneous fat thickness and fat thickness taken at three different positions over the *Musculus longissimus dorsi* (the rib-eye muscle); pH_24_; carcass lean; carcass external length (CEL); buttock circumference (BC); and rib-eye width. Treatment diets affected (*P* < 0.05) rib-eye area (REA). Lambs on treatments T1 and T5 had lower (*P* < 0.05) rib eye area (REA) than those on T4. The pH_1_ values were influenced (*P* < 0.05) by treatment. Lambs on treatment T5 had higher (*P* < 0.05) pH_1_ values than those on other treatments.

## Discussion

### Chemical composition of treatment diets

It is a general practice to include a minimum amount of roughage in high-concentrate feedlot diets to maintain rumen health and reduce digestive disorders (Mertens 2002). According to Jolly and Wallace (2007), ruminants appear to differ in their minimum fibre requirements; sheep in particular require a minimum of 10% roughage in the diet. However, in this study, all diets contained 40% roughage from different sources. Mertens (2002) cautioned against feeding an excessive amount of fibre because it can increase rumen fill and reduce the dry matter intake (DMI), which subsequently also reduces animal growth.

In this study, the NDF contents of the diets were 50% or below and was lower than the concentration suggested (60—65%) to limit intake and digestibility of nutrients in ruminants (Van Soest et al. 1991). Similarly, Van de Vyver et al. (2014) also used feedlot diets for lambs with the NDF contents ranging from 24.3–42.1%, where Lucerne hay was replaced with different inclusion levels of maize silage as a roughage source. The NDF contents of the five treatment diets used in this study (Table 2) were, however, higher than the recommended range of 15 to 20% NDF by Smith (2008).

### Feed intake

Feed intake is a major factor that influences the amount of nutrients available to the lamb in order to realise its growth potential (van der Merwe et al. 2020). A key concern of high proportions of roughage in feedlot rations is the high NDF content, which may physically restrict the dry matter intake through rumen fill (Jolly and Wallace 2007; Oba and Allen 1999). Intake is a function not only of the NDF concentration, but also of the source of fibre (Ruiz et al. 1995), which partly explains the variability in intake of diets formulated from different roughage sources. Notwithstanding the 2-week adjustment period, the feed intake increased from week 1 to 6 for all treatments, which may indicate a gradual adjustment of the rumen microbes to the diets. Hence animals may require a longer adjustment period (6—8 weeks) in feeding trials involving highly fibrous diets (40%) as implemented in this study. Mapiye et al. (2009) also suggested a longer adjustment period for cattle supplementation diets formulated using *Acacia karroo* leaf-meal due to the low diet palatability. Peak ADFI was attained earlier at 6 weeks for T1 compared to 7 weeks for T2 – T5, which may be explained by the higher ADFom and ADLom in bush-based diets requiring a longer adaptation period for the rumen microbes.

The ADFI was affected by treatment * week interactions, which may be partly due to the inevitable variation in the supplied feed caused by natural variability by batch in the ingredients particularly for the bush material, which was harvested from different farms and over varying terrain and soils that ultimately may affect the physical and chemical characteristics of the diets. Flores-mar et al. (2018) replaced alfalfa hay with sorghum straws as roughage source and obtained similar dry matter intake when diets were formulated to contain the same percentage of forage NDF. This was not the case in the current study, as the diets were only formulated to contain similar inclusion levels of roughage sources but not the NDF content.

The digestibility coefficients for DM, OM, CP, NDFom and ADFom for T1 generally exceeded those for other treatments (Shiningavamwe 2022), which may have contributed to higher ADFI for T1 compared to the bush-based diets. Treatments T4 and T5 had lower NDFom compared to T2 and T3, which possibly contributed to a faster rate of digestion, hence reducing rumen fill. Other factors including high palatability and high passage rate (Jolly and Wallace 2007) may have contributed to the greater intake of T4 and T5 than T2 and T3. Worth noting is the considerable feed wastage due to the sorting behaviour when feeding lambs observed during the trial, which was solved earlier in the trial by addition of water (200 ml) to the feed, which helped in binding the feed ingredients, hence reducing the sorting and selection of feed components.

### Growth performance

The non-significant contrasts of body weights for sheep on the control diet versus the rest of the diets across the 13 weeks, is surprising given that bush-based diets on account of their higher ADFom and ADLom (Table 2), should have restricted intake and hence body weight gains. This result, however, implies the equivalence of bush-based diets to grass hay in conventional diets in serving as a roughage source for moderate live weight gains. Superior performance on T2 compared to other diets may be attributed to the higher metabolizable energy content, high nutrients digestibility and a better amino acid profile (Shiningavamwe 2022).

Unlike the body weights which were affected by treatment x week and treatment x sex interactions, ADG and FCR are summary measures that provide aggregate performance and may mask the inherent fluctuations and subtle differences among animals under different treatments. The differences in growth rates of male and female lambs observed in this study among the different treatment diets were similar to the observations by Rodríguez et al. (2008) in a fattening study on Assaf lambs, which was partly associated with dietary selection by animals of different sex. These authors found that the growth rates were lower in females than in males which were linked to similar trends in their dry matter and crude protein intake.

According to van der Merwe et al. (2020), for profitable production, producers often aim for an ADG of 300 g/day and FCR of 5.0 kg feed/kg weight gain, depending on the breed and type of feed used in the finishing system. In this study, the best ADG (156.4 ± 6.9 g/day) and FCR (7.9 ± 0.5) were obtained with T2, which were far more inefficient than the proposed figures above. The poorer performance for ADG and FCR in the present study could be related to the high inclusion rate of low-quality roughage sources than most conventional feedlot diets, which usually contain 20% or less roughage. Ismail and Obeidat (2023) who used Awassi lambs with a 25% roughage diet of wheat straw and olive leaves obtained ADG in the range of 218 to 235 g/day and FCR in the range 4.19 to 4.52. Van de Vyver et al. (2014) who replaced lucerne hay with maize silage as roughage source, reported similar FCR to T1 and T2 in this study. As indicated above, however, this level of FCR is not optimal for the production of sheep in a feedlot. Therefore, future research is warranted to optimize these diets to specification levels for growing lambs in the feedlot, with special focus on inclusion levels of each encroacher species depending on their physical and chemical fibre characteristics. There may also be a need to chemically or enzymatically treat bush-based feeding material so as to improve nutrient utilization (Adesogan et al. 2019).

### Carcass characteristics

#### Carcass classification

Carcass classification systems were developed to inform processors and consumers about the quality of the carcass and thus to distinguish its market value (Brand et al. 2018). In the present study, all carcasses of the Damara lambs in the five treatment diets fell in the same age category (A: with no permanent incisors) and fatness score 1, which gave an overall A1 grade. The age category was expected to be similar since they were all born within the same lambing season. The A1 grading could be explained by the fact that Damara sheep are fat-tailed and much of the body fat is accumulated around the tail with minor fat deposits in the rest of the body (Kleemann et al. 2000; Tshabalala et al. 2003; Almeida 2011; Wilkes et al. 2012; Almeida et al. 2014). Therefore, this implies that the milled bush diets are good enough to produce lean meat.

#### Carcass measurements

Final, hot carcass and cold carcass weights were heavier in males than females which is consistent with findings by Simela et al. (2011) in goats and Van der Merwe et al. (2020) in lambs. Dressing percentage was in the range 37.4 to 40%, which is similar to what was reported in Menz sheep (Assefa et al. 2008), but this falls far short of dressing percentage reported in other studies for example: 46.1–48.3% (Cardoso et al. 2021); 49.4–50.2% (Rezaei et al. 2013); 59.9% (Simela et al. 2004). Dressing percentage is affected by live weight, fatness, time off water and feed, sex and breed (Warmington and Kirton 1990). Higher growth rates have been typically associated with higher dressing percentages (Seoni et al. 2018) and given that ADG in this study was in the range 124 to 156 g/day, the slower growth could have contributed to the lower dressing percentage. Wilkes et al. (2012) reported dressing percentage in Damara sheep of 53.2 ± 1.9%, which reflected greater fatness compared to Merino (dressing percentage = 41.5 ± 1.8%). Results of this study suggest that while bush-based diets may be able to meet maintenance requirements, the inclusion rate used in this study might be high for supporting optimal growth in feedlots. Further studies are needed to determine optimal inclusion levels of browse material in feed rations and to determine whether chemical or biological treatment may improve their utilization.

The pH_1_ for T5 was slightly higher compared to other treatments which may be attributed to low muscle glycogen reserve possibly due to low energy supply (Vestergaard et al. 2000). Although the diets were formulated to be iso-energetic, diet T5 had the lowest DM and OM digestibility (Shiningavamwe 2022) which could have contributed to low energy reserves. It was, however, observed that the ultimate pH_24_ was similar for the different diets. Dietary energy intake impacts muscle glycogen reserves (Daly et al. 2006; De Brito et al. 2016) and these in turn affect muscle pH values. This implies that the observed similar ultimate pH could be attributable to a different factor not muscle glycogen reserves as influenced by dietary energy levels. Muscle pH values affect colour, water-holding capacity and sensory attributes of lamb meat (Ferguson and Gerrard 2014). In this study muscle glycogen reserves were not determined and neither was the eating quality assessed but these would be useful in future studies to determine effects of different bush-based diets on meat sensory attributes.

Rib eye area (REA) is associated with the amount of muscle in a carcass and is indicative of muscle development and yield of high value cuts (Williams 2002). The results indicate greater muscle development for animals on diets T2, T3 and T4 compared to T1 and T5; similarly rib eye depth was greater in T4 than T1. Even after adjusting for differences in CCW, the advantage of T4 over T1 and T5 were still evident for both rib eye depth and REA. It is not clear why the T4 diet could have positively influenced muscling without impacting ADG.

## Conclusions

The milled bush of the four-encroacher species can be used to partially replace grass and Lucerne hay in diets for weaned lambs. Lambs fed T2 had higher ADG and better FCR than other treatment groups, but still fell short of targets for commercial sheep feedlots, probably due to low nutrient digestibility of the bush roughage sources. The lambs fed the bush-based diets had carcass characteristics that were comparable to those on the control diet. Cold carcass weights, grading, ultimate pH and fat thickness were similar among treatments. Although encroacher bush could be used as an alternative roughage source, the low dressing percentage possibly related to slow growth rates is of particular concern because it impacts profitability. Therefore, more research is needed to develop efficient feeding strategies or processing such as pelletizing, that encourage inclusion of milled bush as a roughage source for ruminants.

## Data Availability

The datasets generated for the current study are available from the corresponding author on reasonable request.
